# Microbiome modulation, microbiome protein metabolism index, and growth performance of broilers supplemented with a precision biotic

**DOI:** 10.1016/j.psj.2023.102595

**Published:** 2023-02-16

**Authors:** Cristiano Bortoluzzi, Ian Tamburini, Jack Geremia

**Affiliations:** ⁎ DSM Nutritional Products, LLC, Kaiseraugst, Switzerland; † DSM Nutritional Products, LLC, Lexington, MA, USA

**Keywords:** broiler, microbiome metabolism, precision biotic, metagenome

## Abstract

The objectives of the present studies were to evaluate: 1) the *in vivo* impact of the supplementation with a precision biotic (**PB**) on the growth performance and microbiome modulation of broiler chickens; 2) the role of PB on the modulation of functional pathways of the microbiome collected from animals with low and high body weight gain, and 3) to develop a Microbiome Protein Metabolism Index (**MPMI**) derived from gut metagenomic data to link microbial protein metabolism with performance. The *in vivo* work consisted of 2 experiments with 2 treatments: Control vs. PB at 1.1 kg/MT of PB with 21 or 14 replicates of 40 birds per replicate, in experiments 1 and 2, respectively. Growth performance was evaluated in both experiments, and from experiment 1, cecal samples from one bird/replicate was collected on d 21 and 42 (*n* = 21/treatment) to evaluate the microbiome through whole genome sequencing. In the *ex vivo* assay, 6 cecal samples were collected from low body weight (**BW**) birds (at 10% below average), and 6 samples from high BW birds (at least 10% above average). The samples were incubated in the presence or absence of PB. After incubation, DNA was isolated to develop a functional genomic assay and the supernatant was separated to measure short-chain fatty acid (**SCFA**) production. The MPMI is the sum of beneficial genes in the pathways related to protein metabolism. In the *in vivo* grow out experiments, it was observed that the supplementation improved the BW gain by 3% in both studies, and the corrected feed conversion ratio (**cFCR**) by 3.7 and 3.4% in studies 1 and 2, respectively (*P* < 0.05). The functional microbiome analysis revealed that the PB shifted the microbiome pathways toward a beneficial increase in protein utilization, as shown by higher MPMI. In the *ex vivo* experiment, the PB increased the abundance of genes related to the beneficial metabolism of protein (quantitative MPMI), and the concentration of SCFA, regardless of the underline BW of the birds. Taken together, the microbiome metabolic shift observed in the *in vivo* study and higher MPMI, plus the observations from the *ex vivo* assay with higher SFCA production, may explain the improvement in growth performance obtained with the supplementation of PB.

## INTRODUCTION

The poultry intestinal microbiome is the community of microorganisms that resides within the intestine. It acts as a functional organ that, among other mechanisms, interacts metabolically with the host bird and can be characterized by its metagenome, which is the collection of genetic material drawn from across the entire microbial ecology. Among other functions, the gut metagenome encodes for a variety of microbial pathways that convert unabsorbed nutrients and components from the feed into physiologically and environmentally consequential microbial metabolites (Sergeant et al., 2014; Fan et al., 2015; Glendinning et al., 2020). Unlike phylogenetic composition (e.g., the taxonomy or microbial profile of the gut microbiome), which is known to vary widely between otherwise identical birds, various metabolic functions of the intestinal microbiome are stable between different hosts (Turnbaugh et al., 2007; Oakley et al., 2014; Jiang et al., 2016; Proctor et al., 2019). It has been suggested that this stability results from the fact that many phylogenetically diverse microorganisms harbor similar genes capable of similar functions (Tian et al., 2020). There has been growing interest in harnessing the full potential of the intestinal microbiome, via the core metabolic functions it performs, for improving nutritional health, performance, welfare, and sustainability in commercial poultry production (Huang et al., 2018; Yadav and Jha, 2019).

In production animals the microbiome has been shown to play an important role in pathogen protection (Clavijo and Flórez, 2018), immune system development (Crhanova et al., 2011) and nutrient production (Józefiak et al., 2004). Most of the benefits originated from the microbiome come from the metabolites that the microorganisms are producing and that are absorbed by the host. Indeed, more than 30% of the metabolites in the bloodstream originates from the microbiome (Chernevskaya et al., 2020), and have critical role in linking digestive and fermentation processes in the gut with animal physiology and health. An important aspect to be observed is related to nitrogen metabolism and protein utilization by the intestinal microbiome that can influence the amount of nitrogen excreted into the environment. Nitrogen can reach the hindgut in 2 ways, either via overspill from the small intestine or via endogenous losses (Reese et al., 2018). From the dietary origin under healthy conditions, most protein is digested and absorbed in the small intestine; however, overfeeding or low quality protein can lead to an overflow of undigested protein that reaches the distal portions of the gastrointestinal tract (**GIT**). Furthermore, reduced intestinal barrier function, manifested as a breakdown in tight-junction integrity and ultimately intestinal lesions, may increase the leakage of plasma protein into the intestinal lumen and lead to an excess of undigested protein in the hindgut. Reduced barrier function often results from parasitic conditions such as coccidiosis, bacterial necrosis, and even from the damaging biochemical action of microbial metabolites such as ammonia and hydrogen sulfide on the epithelial layer (Yokoo et al., 2021).

Nondigested protein may be metabolized by certain functional bacterial groups, such as putrefactive or protein-fermenting bacteria, that will produce toxic end-products (Apajalahti and Vienola, 2016). The presence of branched-chain fatty acids (**BCFAs**) in the cecal content of chickens, for example, indicates that protein fermentation is ongoing. Although BCFAs are not known to be toxic, other metabolites, such as 3-methyl-indole (skatole), ammonia, and high concentration of biogenic amines may be detrimental for the health of animals (Apajalahti and Vienola, 2016). Therefore, a deeper understanding of how the microbiome controls protein metabolism and utilization is needed in order to develop novel approaches that will redirect microbial pathways toward a less putrefactive metabolism.

A novel precision biotic (**PB**) that modulates core microbiome metabolic pathways related to nitrogen metabolism and short-chain fatty acid (**SCFA**) production, beyond the microbial profile, regardless of the geographic region, dietary composition, season, bird genetic, among others, has recently been presented ( Walsh et al., 2021; Jacquier et al., 2022). The PB used herein is a mixture of chemically designed glycans that specifically modulate, or signal, the bacterial DNA to perform desirable functions mainly related to nitrogen metabolism and SCFA production (Walsh et al., 2021; Jacquier et al., 2022). These aspects would positively impact health, welfare, growth performance, and environmental sustainability. The hypothesis of the present studies was that the PB would modulate microbiome metabolic pathways toward a better metabolism and utilization of protein and improve the growth performance of broiler chickens. Additionally, it was hypothesized that the PB could modulate, *ex vivo*, microbiome metabolic pathways from animals with varying body weights (**BWs**). Therefore, the objectives of the present studies were to evaluate: 1) the *in vivo* impact of supplementation with a PB on the growth performance and microbiome modulation of broiler chickens; 2) the role of PB on the modulation of functional pathways of the microbiome collected from animals with low and high body weight gain (**BWG**), and 3) to develop a Microbiome Protein Metabolism Index (**MPMI**) derived from gut metagenomic data to link microbial protein metabolism with performance.

## MATERIALS AND METHODS

### Animals, Housing, and Experimental Design

Animal trials were conducted in accordance with the Guide for the Care and Use of Agricultural Animals in Agricultural Research and Teaching (FASS, 2010), and followed by qualified personnel.

Chicks were obtained from a commercial hatchery. In Experiment 1 a total of 1,680 one-day-old male broilers chickens (Hubbard Cobb) were randomly divided into 2 treatments, with 21 replicate pens per treatment and 40 birds per pen according to a completely randomized block design. In Experiment 2, 1,120 one-day-old male broilers chickens (Hubbard Cobb) were randomly divided into 2 treatments, with 14 replicate pens per treatment and 40 birds per pen, according to a completely randomized block design. Only animals free of any clinical signs were included in the trial. In both trials, the experimental treatments consisted of 2 basal diets that differed in the PB inclusion (Table 1). The treatment groups were a basal diet without PB, or basal diet supplemented with 1.1 Kg/MT of PB (Symphiome, DSM Nutritional Products, Kaiseraugst, Switzerland). All the birds were vaccinated against coccidiosis, Marek and Newcastle disease at the hatchery.

**Table 1:** Feed formulation and calculated composition of the feed used in Experiments 1 and 2.

Ingredients, %	Starter	Grower	Finisher
Corn	58.35	65.38	69.41
SBM, 47.9% CP	29.99	20.40	16.88
Fat	0.05	0.05	0.05
Wheat middling	2.38	0.34	0.12
DDGS	5.00	10.00	10.00
Salt	0.342	0.332	0.332
DL-methionine	0.515	0.4.77	0.420
L-Lysine-HCL	0.367	0.439	0.400
Limestone	1.794	1.537	1.470
Dicalcium phosphate	1.079	0.869	0.739
Choline chloride—70%	0.044	0.080	0.091
Mineral and vitamin premix^1^	0.075	0.075	0.075
Ronozyme HiPhos GT	0.010	0.010	0.010
Calculated composition			
Protein, %	20.3	17.5	16.0
ME kcal/kg	2,900	3,040	3,084
Ca, %	0.920	0.770	0.720
Av. P, %	0.350	0.300	0.270
Sodium, %	0.160	0.160	0.160
Dig Lys, %	1.330	1.140	1.010
Dig M+C, %	0.990	0.880	0.790
Choline, g/kg	1.600	1.600	1.600

1 The premixes included vitamins A, D3, E, K3, and B complex vitamins along with manganese, iron, zinc, iodine, copper, selenium, and calcium at commercially relevant levels.

Feed and water were supplied *ad libitum* throughout the experiment. The birds were reared under a standard poultry industry starter diet (0–10 d; crumble), grower diet (11–24 d; pellet) and finisher diet (25–42 d; pellet) in floor pens with new litter. Diets were based on corn-soybean meal, with inclusion of DDGS and wheat middling, formulated to meet or exceed the breeders’ recommendations, except for metabolizable energy (100 kcal lower); all the diets had inclusion of phytase (1,000 FYT/kg; RONOZYME HiPhos GT, DSM Nutritional Products; Table 1). The PB used in these trials has been previously described by Walsh et al. (2021), and Jacquier et al. (2022).

Feeders were checked daily to ensure that all the birds had access to feed. Lighting program made use of incandescent lighting for approximately 23 h of continuous light and 1 h of darkness per day for d 0 to 7, and for approximately 20 h of continuous light and 4 h of darkness per day for the remainder of the study. BW and feed intake (**FI**) were collected on d 0, 10, 24, and 42 during the growing period. BWG, FI, and corrected feed conversion ratio (**cFCR**) as well as the European Poultry Efficient Factor (**EPEF**) were calculated for 0 to 42 d. Corrected FCR was corrected by the target BW at 42 d of the specific strain of broiler used. EPEF was calculated by the following formula: EPEF = (average daily gain)/(mortality corrected FCR*10)*(100% of mortality).

### Sample Collection

In experiment 1, on each of the 2 sampling days, d 21 and 42, one (1) bird was selected randomly from each pen (21 pens per treatment). Ceca were removed from each sampled bird, and the cecal contents were quickly aliquoted into 5 mL conical tubes with cryo-safe labels identifying the source bird. Tubes were sealed and immediately frozen on dry ice. Samples were maintained at −80°C and thawed immediately prior to DNA extraction. The BW of the control group sampled birds was recorded, and the samples were grouped into cohorts as either: Low BW or High BW, based on whether its corresponding bird weight was below or above the mean, respectively.

### DNA Extraction, Sequencing, and Functional Mapping

Microbial DNA was extracted from 200 µg of cecal sample by using Qiagen DNeasy PowerSoil HTP96 kit (Qiagen, Düsseldorf, Germany), following manufacturer protocols. DNA was sequenced on an Illumina HiSeq 3000 instrument, with a target depth of 5GB per sample. Raw fastq files from shallow shotgun sequencing were inspected using FastQC v0.11.5. Based on the quality reports, Cutadapt was used to trim the first 10 bases of each read, shorten each read to a maximum of 130 bp, and discard any reads below 120 bp. Processed reads were mapped against an internal gene catalog expanded from that of Glendinning et al. (2020) curated for the chicken intestinal microbiome with bwa v0.7.5 using the BWA-MEM algorithm (Li and Durbin, 2009, 2010; Li, 2013). Gene counts were extracted for each sample from BAM and annotated using the publicly available KEGG Orthology (**KO**) database (Kanehisa and Goto, 2000). KEGG Ortholog gene counts were then further annotated by Enzyme Commission (**EC**) Number using the KEGG reaction database. The reaction-annotated gene count data were summed and regrouped by EC number, and then internally normalized per each sample, using the total gene abundance of the “glycolysis/TCA Cycle” KEGG Module and the normalization denominator. The resulting data file contained, for each sample, the normalized abundance of 2,083 EC numbers, annotated by the top 5 KEGG pathways for which that EC number was known to participate.

### Functional Metagenomic Profiling

Computed functional metagenomic profiles were obtained as follows. For each sampling day, a random forest classifier model was constructed with the objective to discriminate between birds in the Control vs. the PB treatment group. The random forest models were constructed in the R programming language (R version 4.1.2) using the package “randomForest” (R version 4.7-1.1) with dimensionality *mtry* = 2 and number of trees = 10,000. The model features for each sample were provided by the normalized EC number abundances described above. Following construction of the initial random forest model, the EC number model features were ranked in descending order of importance based on the “Mean Decrease in Accuracy” score obtained from the model training. To minimize the potential for model overfitting, the ranked list of EC number features was then truncated to the first 30 (most important) features and the random forest model was retrained on the truncated feature list using otherwise identical random tree parameters. Models were further evaluated for robustness by generating an ensemble of models with different random seeds and small variations in parameters, each trained on different random subsets of the data (e.g., 60% of the available data points for each independent training). Model validation was performed by testing the performance obtained for data points not used explicitly for training.

Top microbial metabolic reactions (EC numbers) and KEGG pathways responsible for distinguishing PB treated birds from Control were identified by sorting EC numbers by “Mean Decrease in Accuracy” using the truncated random forest classifier. Each EC number was then annotated and regrouped by KEGG Pathway. Functional metagenomic clustering was performed by local Fisher discriminant analysis (**LFDA**), and a graphical representation of metagenomic similarity was obtained by plotting each microbiome on a 2-dimensional plane by rank-truncating its LFDA transformed EC number abundances to obtain coordinates.

### Microbiome Protein Metabolism Index

Functional metagenomic features related to microbial nitrogen and protein utilization were selected as a basis for quantifying microbial protein metabolism. These marker reactions, denoted by their corresponding EC number, KEGG pathway identification (Kanehisa and Goto, 2000) and exemplary microbial genes and enzymes, described in Table 2. We defined a MPMI for the microbiome of each bird, calculated as

$$\mathrm{MPMI}=\;100\;\times a\left(\sum_{i}C_{i}\left(N\right)\;/\;\sum_{i}C_{i}\left(D\right)\right),$$

where the *C_i_*(*N*) are the normalized gene counts of numerator reactions (marked “*N*” in Table 2), *C_i_*(*D*) are the normalized gene counts of denominator reactions (marked “*D*” in Table 2), and a is a normalization constant used to account for reaction stoichiometry.

**Table 2:** Metagenomic features used for measuring microbial protein metabolism and calculating the Microbial Protein Metabolism Index (MPMI) in broiler cecal microbiomes.

EC number	KEGG IDs	Role in MPMI^1^	qPCR marker	Representative microbial genes	Representative microbial enzymes
1.2.1.5	K00129	N		ALDH3	Aldehyde dehydrogenase (NAD(P)+)
1.2.7.7	K00187, K00188, K00186	N		vorB, vorD, vorA	2-Oxoisovalerate ferredoxin oxidoreductase beta subunit
1.3.1.95	K20143	N		acrC	Acrylyl-CoA reductase (NADH)
1.3.8.15	K24016	N		acdA	Cumarate reductase
2.3.1.109	K00673	N	y	astA	Arginine N-succinyltransferase
2.3.1.9	K00626	N		ACAT, atoB	Acetyl-CoA C-acetyltransferase
2.6.1.19	K00823, K07250	N	y	puuE, gabT	4-Aminobutyrate aminotransferase, (S)-3-amino-2-methylpropionate transaminase, 5-aminovalerate transaminase
2.8.3.1	K01026	N		pct	Propionate CoA-transferase
2.8.3.18	K18118	D	y	aarC, cat1	Succinyl-CoA:acetate CoA-transferase
3.5.1.107	K13995	D		nicF	Maleamate amidohydrolase
3.5.1.116	K18151	D		UAH	Ureidoglycolate amidohydrolase
3.5.1.54	K01457	D		atzF	Allophanate hydrolase
3.5.2.10	K01470	D			Creatinine amidohydrolase
3.5.2.15	K03383	D		atzD	Cyanuric acid amidohydrolase
3.5.3.12	K10536	D	y	aguA	Agmatine deiminase
3.5.3.23	K01484	D		astB	Succinylarginine dihydrolase
3.5.3.9	K02083	D		allC	Allantoate deiminase
4.3.1.29	K17468	N		dgaE	D-glucosaminate-6-phosphate ammonia-lyase
4.4.1.15	K05396	D		dcyD	D-cysteine desulfhydrase
6.3.1.2	K01915	N		glnA, GLUL	Glutamine synthetase
6.3.4.6	K0194, K14541	N		dDUR1	Urea carboxylase, allophanate hydrolase
6.3.5.5	K01956, K01955, K11541, K11540	N		carA, CPA1, carB, CPA2, URA2, CAD	Carbamoyl-phosphate synthase, aspartate carbamoyltransferase, dihydroorotase

1 Index position indicates the role of the corresponding microbial reaction (EC number) in the Microbial Protein Metabolism Index (MPMI). MPMI is calculated as the ratio of the total normalized gene abundance of Numerator reactions (N) to the total normalized gene abundance of Denominator reactions (D). Marker reactions denote EC numbers selected for the targeted qPCR assay to determine the MPMI for ex vivo experiments.

Specifically, reactions appearing in the numerator of the MPMI correspond to metabolic processes associated with desirable microbial protein assimilation described above (e.g., polyamine biosynthesis, BCFA and SCFA synthesis from amino acids, urea cycle injection), while reactions appearing in the denominator correspond to undesirable microbial protein putrefaction pathways (e.g., microbial production of ammonia, hydrogen sulfide, uric acid, etc.). The MPMI is stoichiometrically normalized such that a value of 100 corresponds to a balance between the abundance of “favorable” vs. “unfavorable” microbial protein utilization. A higher value of the MPMI is associated with more beneficial microbial protein metabolism.

### *Ex Vivo* Incubation of Microbiome Samples

Cecal content samples collected from chickens fed the control diet from the *in vivo* study described above (Experiment 1) on d 42 and classified as low (at least 10% below the average BW; *n* = 6) and high (at least 10% above the average BW; *n* = 6) BW were stabilized for handling as 20% w/v aliquots suspended in 15% PBS/glycerol (Sigma-Aldrich, St. Louis, MO) and frozen at −80°C until further analysis. All ex vivo procedures described herein were performed under anaerobic conditions (<8 ppm atmospheric oxygen) in a specialized microbiology anaerobic chamber (Coy Lab Products, Grass Lake, MI). Cecal suspensions were then thawed anaerobically, centrifuged at 6,000 × *g*, supernatants were removed, and resulting pellets were resuspended to form a 1% w/v slurry in a sterile aqueous mixture of: 900 mg/L sodium chloride, 26 mg/L calcium chloride dihydrate, 20 mg/L magnesium chloride hexahydrate, 10 mg/L manganese chloride tetrahydrate, 40 mg/L ammonium sulfate, 4 mg/L iron sulfate heptahydrate, 1 mg/L cobalt chloride hexahydrate, 300 mg/L potassium phosphate dibasic, 1.5 g/L sodium phosphate dibasic, 5 g/L sodium bicarbonate, 0.125 mg/L biotin, 1 mg/L pyridoxine, 1 mg/L pantothenate, 150 mg/L arginine, 150 mg/L methionine, 150 mg/L threonine, 150 mg/L phenylalanine, 150 mg/L tyrosine, 225 mg/L histidine, 75 mg/L glycine, 225 mg/L tryptophan, 225 mg/L valine, 225 mg/L isoleucine, 300 mg/L leucine, 400 mg/L cysteine and 450 mg/L proline; 250 µL of 1% w/v cecal slurry, 100 µL of sterile deionized water (100 µL of 1% w/v of partially hydrolyzed feed extract if performing SCFA analysis), and 150 µL of PB (provided as a sterile 1% aqueous solution) were loaded into a 96-well deep-well microtiter plate. Loaded plates were incubated anaerobically at 37°C for 12 h, after which the well contents were removed and centrifuged at 3,000 rpm for 12 min to pelletize microorganisms. Supernatants were removed for SCFA analysis, and the resulting pellets were used for DNA isolation as described previously and analyzed according to the assays described below. All reagents and materials were degassed under anaerobic conditions for 24 h prior to use.

### Quantitative Microbial Protein Metabolism Index Assay

Given the large number of samples and conditions explored *ex vivo*, we designed a simpler, faster, and less costly assay for assessing the MPMI. Selected marker reactions, as indicated in Table 2, were used as the basis for constructing a targeted qPCR assay to quantify microbial protein metabolism from the microbiome of cecal samples. To account for sequence diversity in the microbial genes that code for a marker reaction, we developed mixed PCR primer sets by considering the set of known gene orthologs across common microbial taxonomic compositions. Briefly, we surveyed the KEGG Orthology (Kanehisa and Goto, 2000) database using the “sequinr” version 4.2-4 (Charif and Lobry, 2007) and “KEGGREST” version 1.30.0 (Tenenbaum and Maintainer, 2022) packages in R (version 4.0.3) to extract sequence variants for each marker reaction shown in Table 2. We then performed manual alignment and priority-ranking against taxa reported as observed in broiler cecal microbiomes. PCR primers were designed using the OligoPerfect primer design tool and primers with melt temperatures in the range of 55°C to 64°C were selected so that the variants could be used in the same PCR program (ThermoFisher Scientific, Waltham, MA). Primers were synthesized, purified by desalting, and prepared to 100 mM concentration in TE/H_2_O (Rapid Oligo Synthesis, Genewiz, Cambridge, MA). For each marker reaction in Table 2, candidate primers were evaluated based on PCR target affinity and melt-curve shape, consistency, and mutual compatibility within our real-time PCR (**qPCR**) program. The 4 highest-quality primer pairs for each marker reaction were selected and combined into a single, blended primer set for that reaction. Blends were obtained by combining equal volumes of the 4 individual candidate primer solutions to yield the sets listed in Table 3.

**Table 3:** Forward and reverse primer sequences adopted for each marker reaction in the targeted functional metagenomic assay.

Marker reaction	Primer ID	Forward primer	Reverse primer
2.3.1.109	Rxn1_1	CGGTGCTGGAGAAAGAAGGT	CAGTTGTGCGGCGGTTAAAA
	Rxn1_2	GATTTTAGCCGCGCCGATTT	TACCTGACCGATGACGTCCT
	Rxn1_3	TGAGCTTTGCACGCTGTTTC	GTTTGCCGAGACTTTGCCAG
	Rxn1_4	TGGTACAACTATCGCGTCGG	GTCCAGGAACAGCGTACACA
2.6.1.19	Rxn2_1	AGAAAGCCAACGATCTGGGG	CGCAGCACGTTGTAATACGG
	Rxn2_2	GTCATGTTTATCGCGCGCTT	TGATGGCGGCGATATCTTCC
	Rxn2_3	AGACGGATTGCTGGCGATAG	CGCAGCACGTTGTAATACGG
	Rxn2_4	TGAAGACGGCGATCACAACA	CGCAGCACGTTGTAATACGG
3.5.3.12	Rxn3_1	CCTCGTGATTTTGGGGCTGA	GCCATCATAATGCCATCGCC
	Rxn3_2	ATCCGTGGTGTGGATTGGAC	CTACGTGGAATGAACCGCCT
	Rxn3_3	CTGGGGATTGAAGTTCGCCT	AGGCATTCGGTGGTGGTAAG
	Rxn3_4	GCCCGCTACCTACGCTAATT	CATCGTTACGCAATGCAGGG
2.8.3.18	Rxn4_1	CAGTGCACAAGAAGCTGCTG	CGTTCAGCAAGAGCCAAAGG
	Rxn4_2	TACCTGGGGAAGATCGACGT	ATCTCAATGGCCTGGGTGTG
	Rxn4_3	CGCATCTACCGCTCCTGATT	GAAGCCCTCTTCAACCTGCT
	Rxn4_4	TAACTGCGCACACCCTGATT	ACAGCATAGTGCCGGTTTCA

To perform the quantitative assay (qPCR), the reaction was prepared in MicroAmp Optical 96-well Reaction Plates (Applied Biosystems, Foster City, CA). One marker reaction was assayed per well by including only one of the corresponding 4 primer pairs combined in equal volume (0.5 uL per primer). In each well, reagents were prepared by combining 10 uL SYBR Select Master Mix (Applied Biosystems, Foster City, CA), 2 uL each of 100 uM forward and reverse primer solutions, 1 uL of 2.5 mg/mL bovine serum albumin solution, and 5 uL of template genomic DNA (20 uL total). Thermocycling was performed using an StepOnePlus Real-Time PCR system (Applied Biosystems, Foster City, CA) set to the “Comparative Ct” experiment setting. The cycling conditions were as follows: an initial 10-min hold at 95°C followed by 40 cycles of denaturation at 95°C for 15 s and annealing at 60°C for 60 s. Melt curves were generated through a hold at 95°C for 30 s followed by a 0.1°C/s ramp up from 65°C to 95°C. Cycle threshold (*C_t_*) lines were determined automatically by the instrument software. For each sample, qPCR analysis was performed in duplicate.

To reconcile samples potentially containing varied amounts of total microbial material and genomic DNA (**gDNA**) abundances, each marker reaction in the targeted assay were normalized to a measure of total bacterial abundance in that sample. Specifically, relative abundances *A_i_* of each marker reaction are calculated as the ratio of gDNA coding for that reaction (as determined by PCR amplification of the primer sets in Table 3) to the total amount of 16s ribosomal DNA measured for that sample using a universal 16s qPCR primer (U16s_F = GTGSTGCAYGGYTGTCGTCA, U16s_R = ACGTCRTCCMCACCTTCCTC).

**Figure 1:**
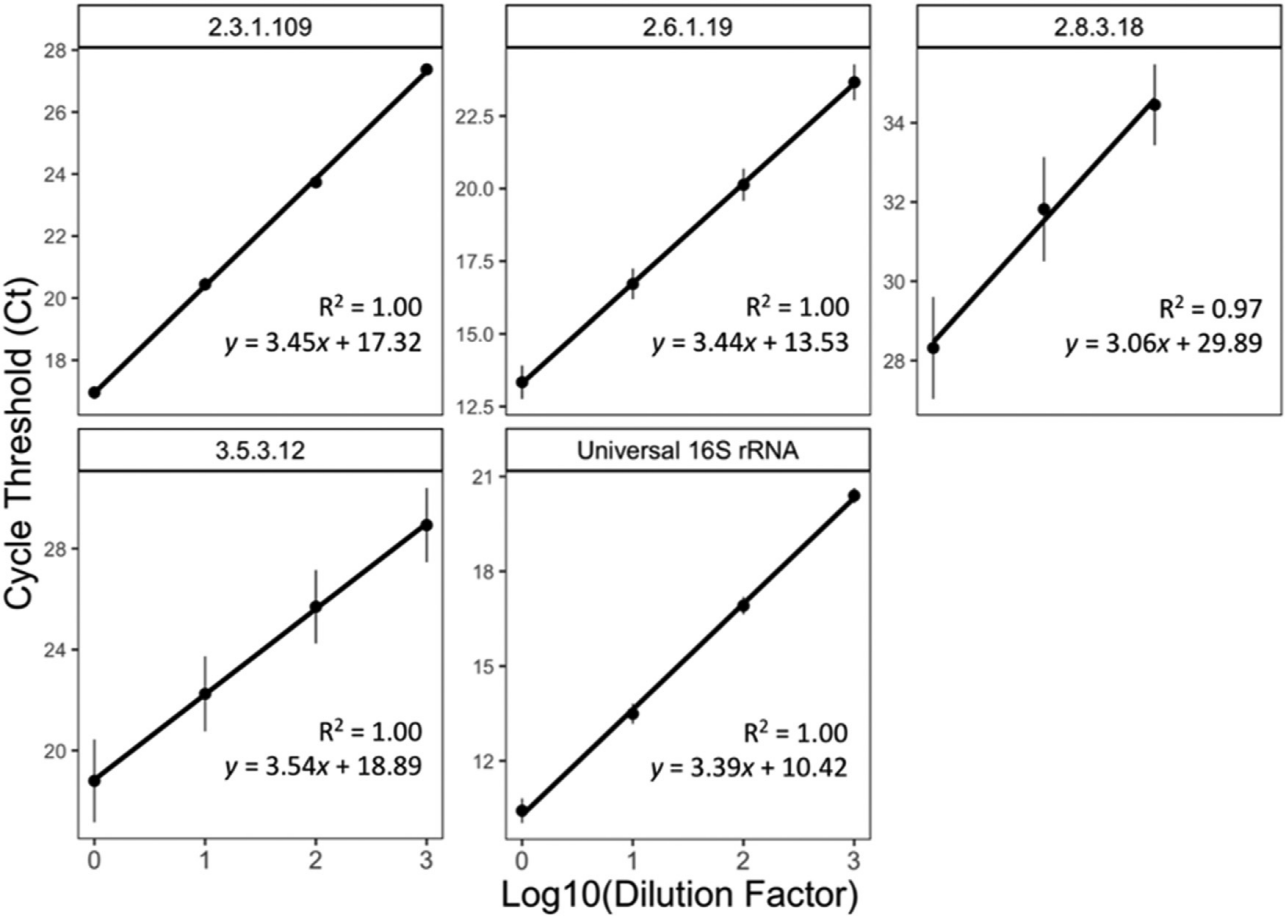
qPCR calibrations for marker reactions obtained from serial dilution of template cecal metagenomic DNA drawn from distinct broiler microbiomes (Experiment 1) across 2 performance cohorts (error bars denote SEM).

Calibration curves for each qPCR primer set were obtained by fitting *C_t_* data obtained from serial dilution of representative cecal metagenomic DNA across 4 orders of magnitude in concentration (approximate range of 0.01–100 ng/uL) to the log-linear model: *Ct_i_* = *m_i_* log_10_[*R_i_*] + *b_i_*, where [*R_i_*] is the amplicon concentration and *m_i_* and *b_i_* are the calibration fit parameters corresponding to each primer set. To assess and minimize source DNA bias, regressions were performed using dilution series obtained from multiple distinct sources of cecal DNA. Regressions were performed using the *lm*() function in R, with the command *lm*(*C_t_ ∼* log_10_(*D*) + factor(*S*)), where *D* is the dilution factor and *S* is a factor that labels the source of the cecal DNA. Calibration results, including regression fits and quality metrics are provided in Figure 1. For each marker reaction, its relative abundance was calculated from the calibration data as follows:

$$A_{i}=\frac{\left[R_{i}\right]}{\left[16s\right]}=10^{\left(\frac{Ct_{i}}{m_{i}}\;-\;\frac{Ct_{16s}}{m_{16s}}\right)}.$$

The qPCR MPMI assay readout was defined as the ratio of total abundances of all marker reactions, calculated as the sum of the relative abundances *A_i_* (after averaging across PCR replicates) over each marker reaction, as specified in Table 2. We refer to the assay measurement as the quantitative MPMI (**qMPMI**) to differentiate from the MPMI measured through full metagenomic sequencing. Statistical comparisons between sample groups (e.g., treatment groups, performance cohorts, etc.) were performed by applying a Mann-Whitney U test (Wilcoxon rank-sum test) implemented using the wilcox.test() function from the “stats” package in R (version 4.0.3) with metagenomic DNA sample as the statistical unit. Effects were considered significant at *P* < 0.05.

### Short-Chain Fatty Acid Analysis from the *Ex Vivo* Supernatants

As further investigation of mode of action consistency of the PB, we performed a targeted metabolomic analysis of SCFA production across BW cohorts. Supernatants were extracted in volumes of approximately 300 uL and analyzed by HPLC. Samples were processed using an Agilent 1100 series HPLC equipped with a refractive index detector (Agilent Technologies, Santa Clara, CA), and were eluted at 40°C at a flow rate of 0.625 mL/min with 0.05% trifluoroacetic acid in water through coupled guard columns (PL aquagel-OH, 50 × 7.5 mm, 5 um guard column (Agilent Technologies) appended with a SecurityGuard Guard Cartridge System (#KJO-4282, Phenomenex, Torrance, CA)), followed by a Rezex ROA-Organic Acid H+ (8%) LC Column 300 × 7.8 mm (Phenomenex). Data were acquired using ChemStation (Agilent Technologies) with SCFA concentrations determined by reference to authentic standards.

### Statistical Analysis

*In vivo* growth performance data were analyzed by 1-way ANOVA (*P* < 0.05) with pen as the experimental unit using JMP 15. *In vivo* and *ex vivo* microbiology, functional metagenomics, metabolism index, and qPCR gene abundance data were analyzed for statistical significance using either the Kruskal-Wallis test by ranks or Mann-Whitney U test (Wilcoxon rank-sum test). Tests were implemented using the stats_compare_means() function from the “ggpubr” package using the R programming language [R version 4.1.2 (2021-11-01)]. Effects were considered significant at *P* < 0.05.

## RESULTS

### Growth Performance

The growth performance results of experiments 1 and 2 are shown in Table 4. We observed a significant improvement (*P* < 0.05) in BWG when birds were supplemented with PB (by 3% in both studies). Additionally, cFCR was improved (*P* < 0.05) by 3.7 and 3.4% in studies 1 and 2, respectively. Consequently, the EPEF was enhanced in studies 1 and 2 (*P* < 0.05) by 5 and 6%, respectively.

**Table 4:** Growth performance of broiler chickens from 0 to 42 d of age fed a control diet or supplemented with a precision biotic.

Treatment	BWG, 0–42 d	cFCR*, 0–42 d	EPEF, 0–42 d
Experiment 1			
Control	2,463^b^	1.855^b^	376^b^
Precision biotic	2,538^a^	1.786^a^	397^a^
SEM	17.59	0.01	6.51
*P* value	0.002	<0.0001	0.03
Experiment 2			
Control	2,393^b^	2.004^b^	358^b^
Precision Biotic	2,464^a^	1.936^a^	381^a^
SEM	18.5	0.01	6.70
*P* value	0.003	<0.0001	0.006

Abbreviations: BWG, body weight gain; cFCR, corrected feed conversion ratio; EPEF, European Poultry Efficiency Index; SEM, standard error of mean.

⁎ FCR corrected for mortality and target BW at 42 d for the specific broiler strain.

a,b Means with different superscripts are significantly different (P < 0.05).

### Microbiome Functional Analysis

**Figure 2:**
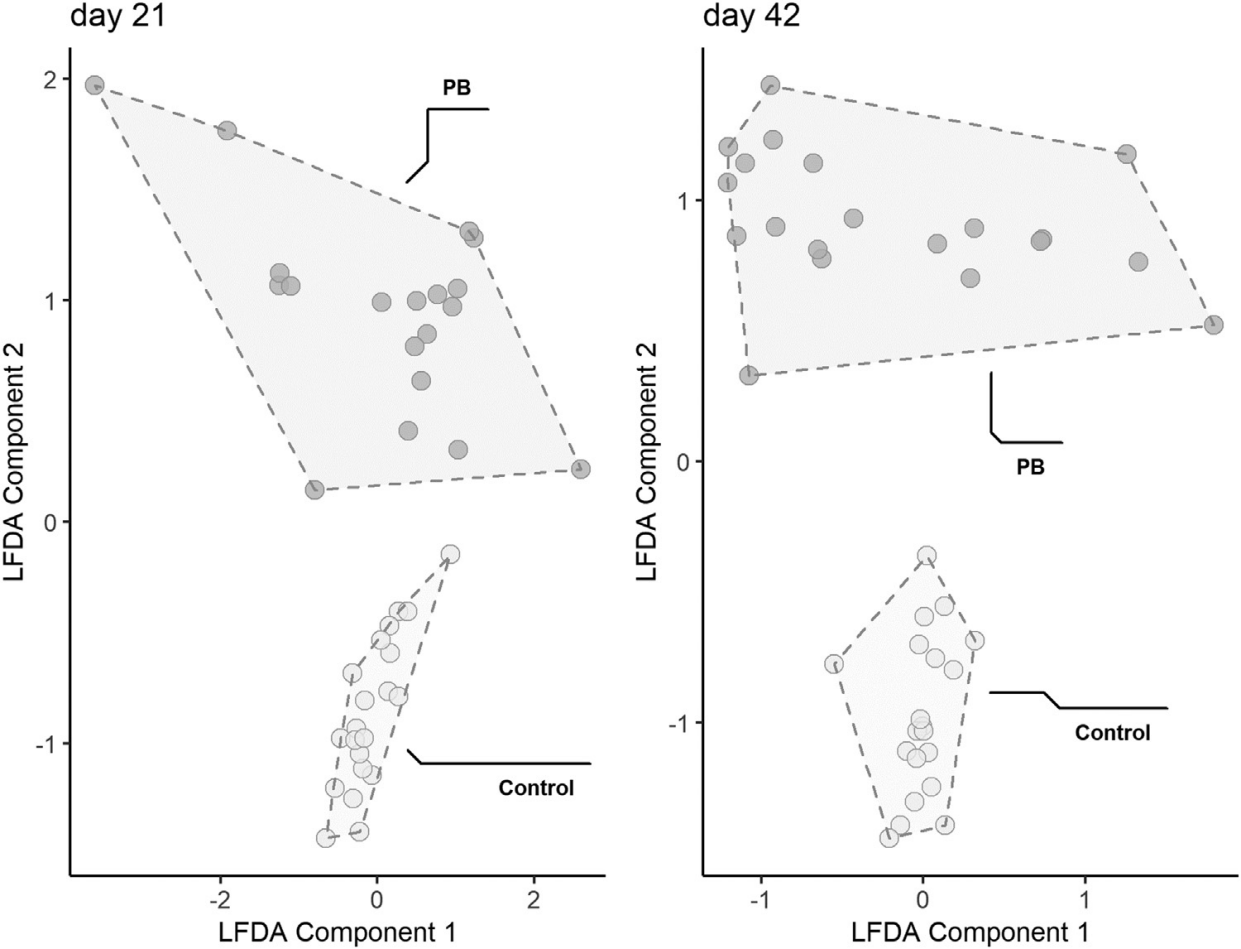
Local Fisher discriminant analysis (LFDA) of functional profiles demonstrating distinct clusters for Control and Precision Biotic fed birds. Abbreviation: PB, precision biotic (Experiment 1).

**Figure 3:**
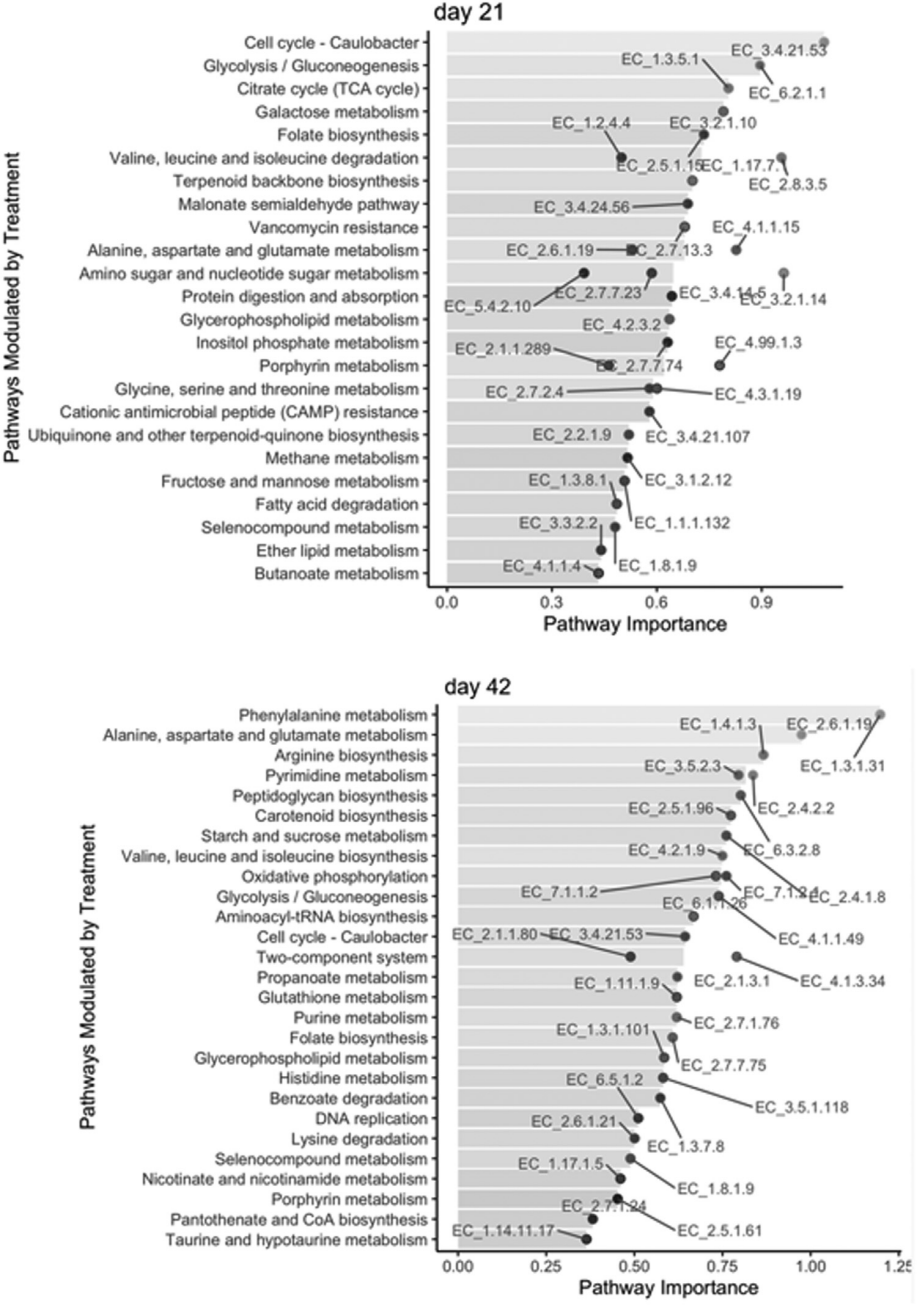
KEGG pathway importance in the microbiome of 21- and 42-days-old chickens supplemented with precision biotic relative to the microbiome of chickens fed the control diet (set as 0). Enzymes (labeled points) were mapped back to KEGG pathways (horizontal bars) (Experiment 1).

The results regarding the functional changes in the cecal microbiome of broilers are shown in Figure 2, Figure 3 and Figure 2, Figure 3. The LFDA of functional profiles (Figure 2) shows a clear and significant shift in the cecal microbiome pathways with the supplementation of PB in both sampling days. Regarding the pathway abundances modulated by PB (Figure 3), it was observed that many pathways were enriched due to the supplementation in both d 21 and 42, mainly related to nitrogen and carbohydrate metabolism.

### Microbial Protein Metabolism Index

**Figure 4:**
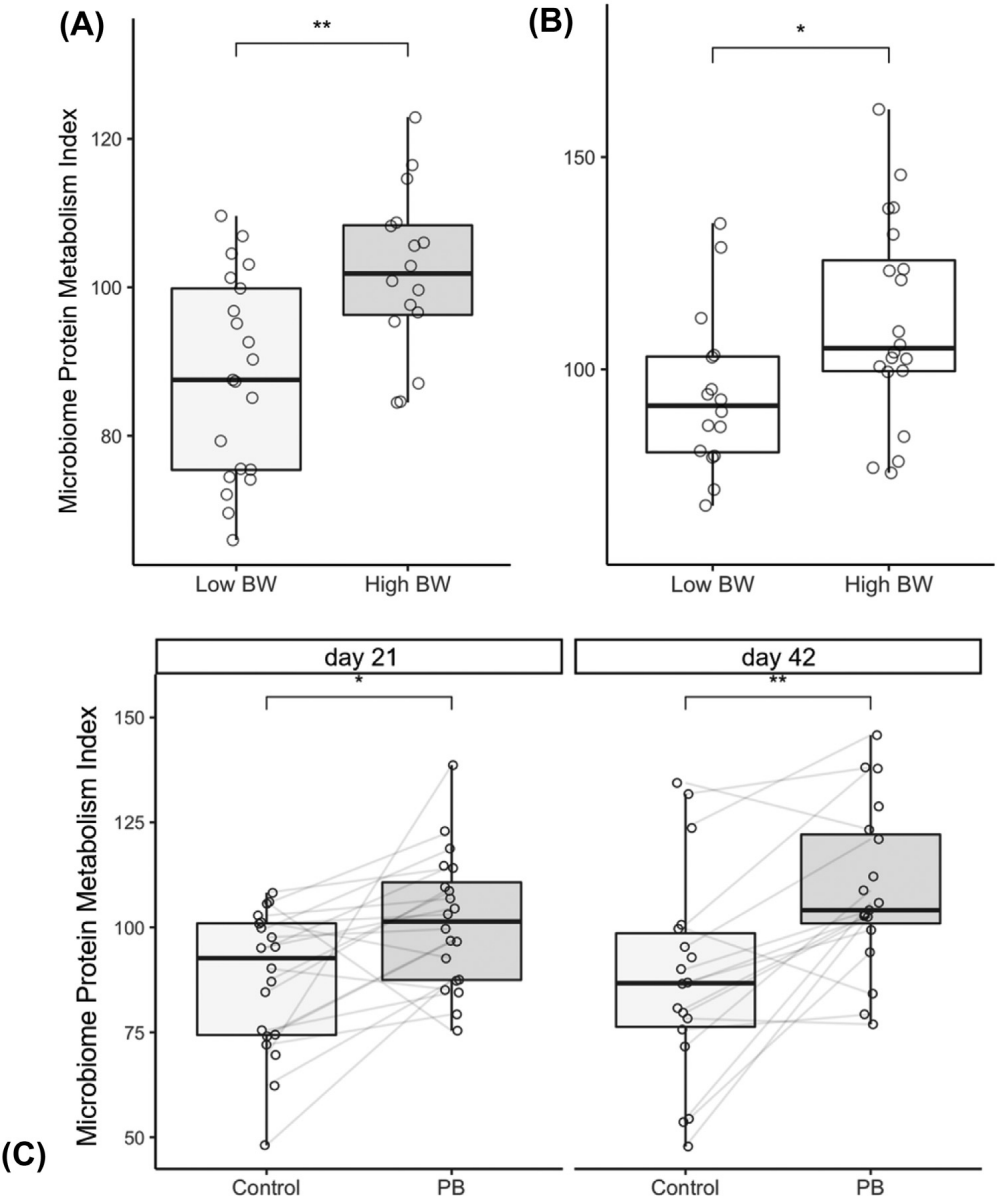
Microbiome Protein Metabolic Index (MPMI) of the cecal microbiome of broiler chickens according to their body weight at d 21 (A) and 42 (B) and supplemented or not with precision biotic (C; PB) at d 21 and 42. The index is increased at both ages with the supplementation of PB (*n* = 21; * *P* < 0.05, and ** *P* < 0.01) (Experiment 1).

The MPMI was calculated for each bird and values were plotted to compare between weight of the animals and treatments on both sampling days. Outlier values outside the interquartile range (2×) were excluded from the analysis. Our results show that MPMI measurements are significantly higher among high BW birds (Figure 4A and B; *P* < 0.01 for d 21 and *P* < 0.05 for d 42), and among birds fed PB compared to control on both sampling days (Figure 4C; *P* < 0.05 for d 21 and *P* < 0.01 for d 42).

### *Ex Vivo* Microbiome Analyses

**Figure 5:**
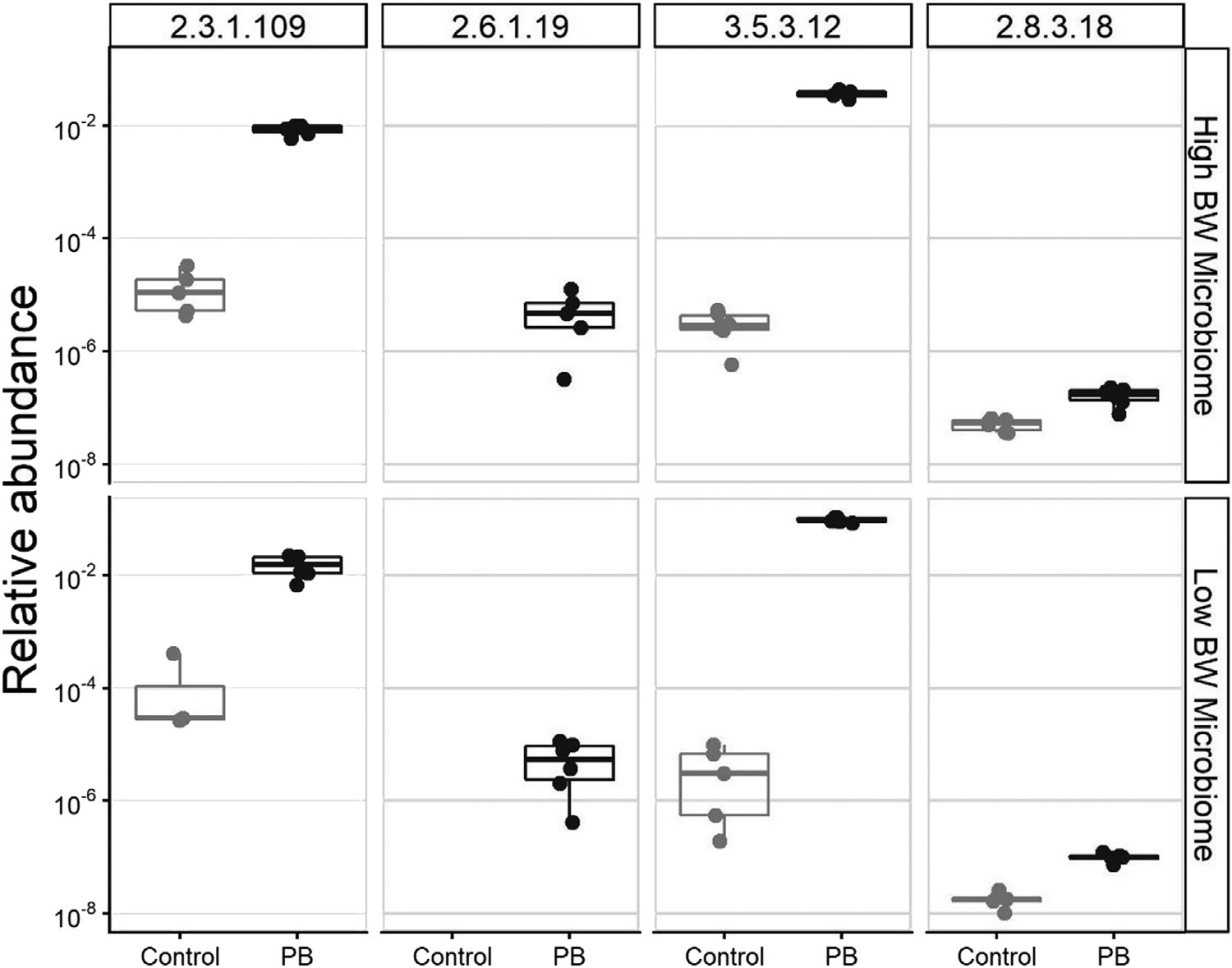
Relative abundances of individual marker reactions (denoted by EC numbers), obtained from the cecal microbiomes of low and high body weight broiler chickens, incubated *ex vivo* with or without the presence of precision biotic (PB). All marker reactions were enriched by treatment with PB (*P* < 0.05).

**Figure 6:**
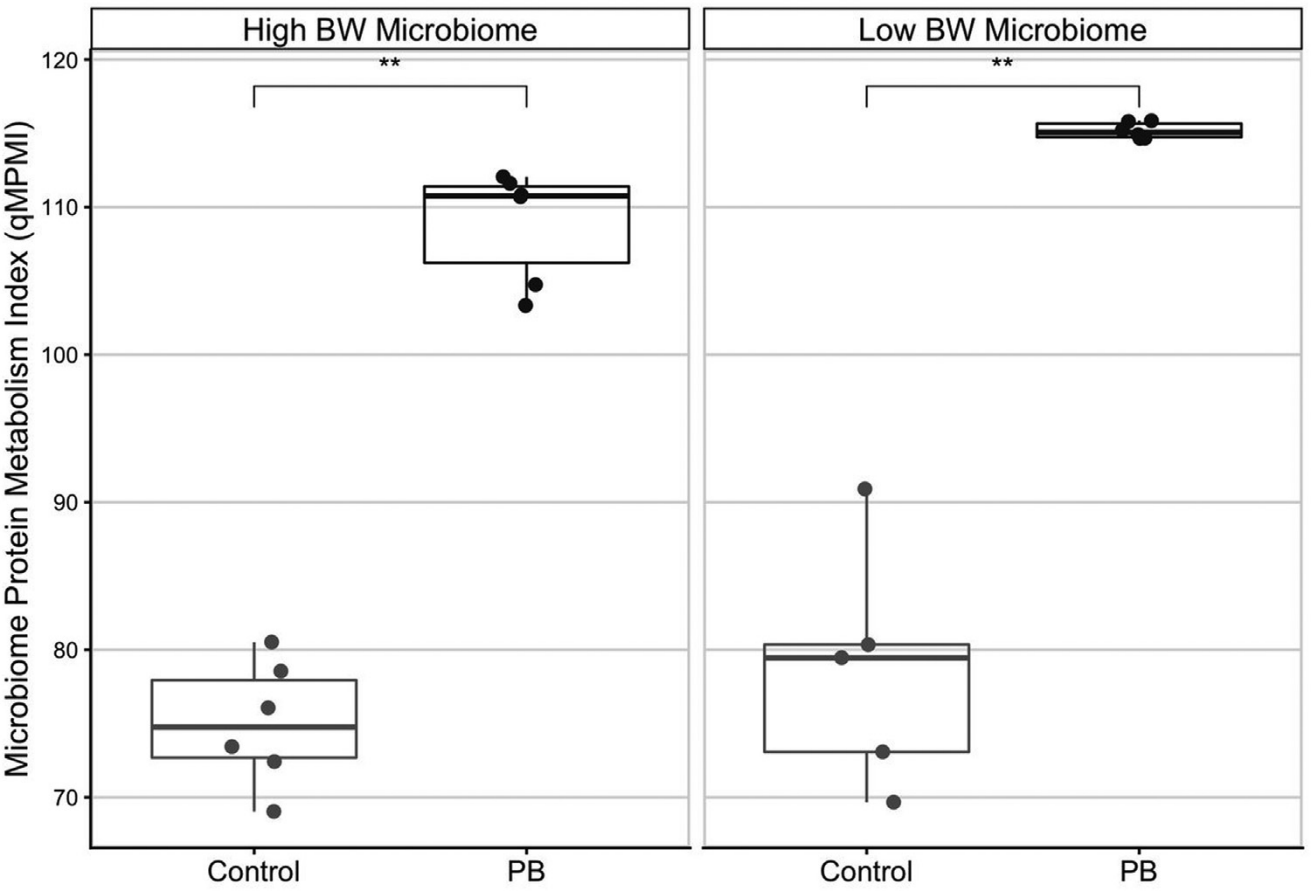
Quantitative Microbiome Protein Metabolism Index (MPMI) of the cecal microbiome of low and high body weight broiler chickens incubated *ex vivo* with or without the presence of precision biotic (PB). The index is consistently increased by PB in both low and high body weight groups (*n* = 6 and *P* < 0.01).

To assess treatment effects of PB on the cecal microbiome corresponding to chickens with different underlying BW, we evaluated the quantitative MPMI of *ex vivo* cecal samples, by evaluating the abundance of individual markers in control vs. PB group (Figure 5). It was observed that the relative abundances were consistently greater by orders of magnitude for the PB treatment group compared to control, regardless of the BW of the animals from which the microbiome sample was collected. Notably, the marker reaction EC 2.6.1.19 was detected exclusively in PB treatment group. Furthermore, it was observed that the presence of PB resulted in an increase of the quantitative MPMI of the cecal microbiome of birds with both low and high BW (*P* < 0.01; Figure 6).

**Figure 7:**
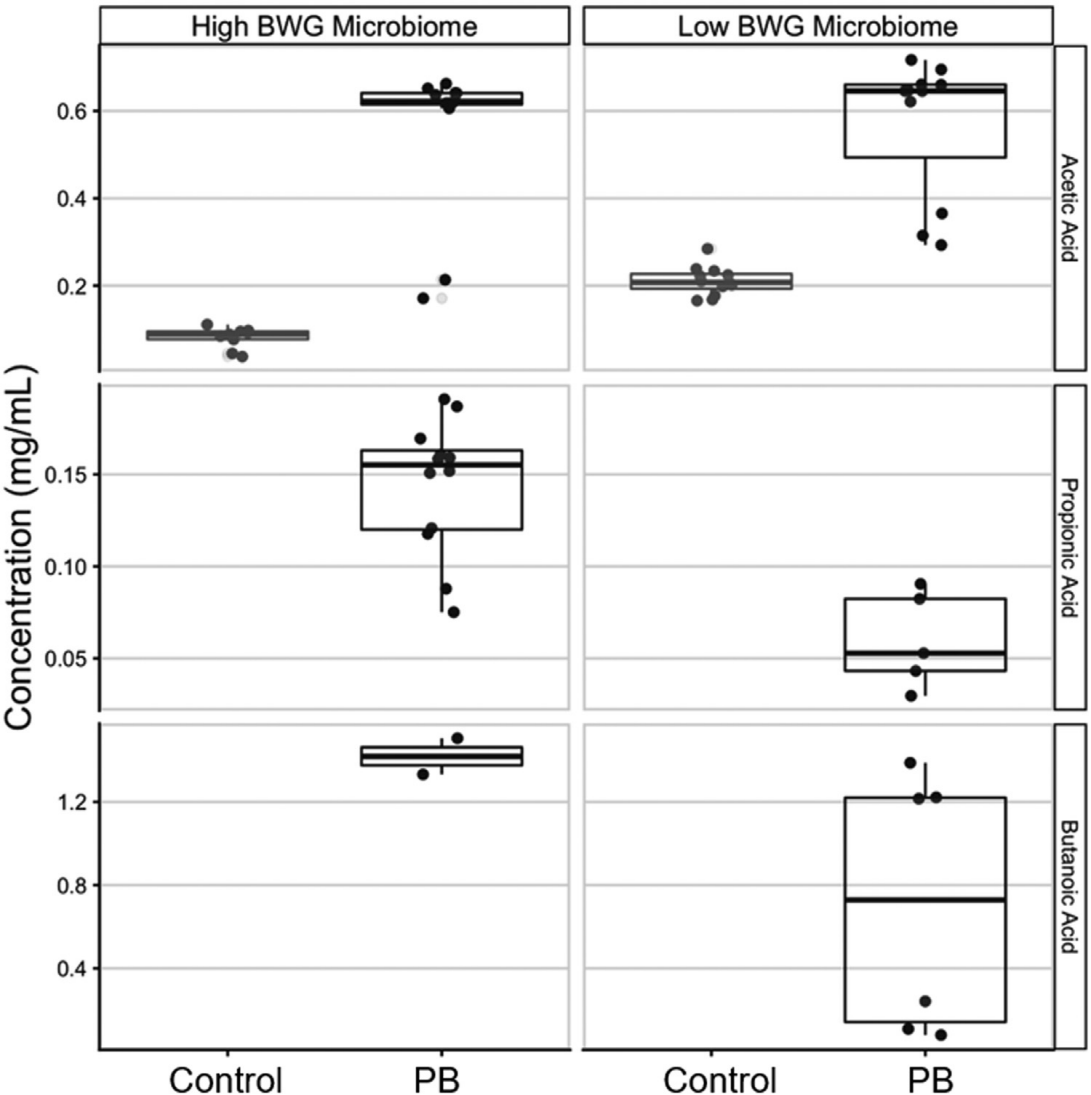
Short-chain fatty acid concentration obtained after *ex vivo* cecal microbiome incubation of low and high body weight birds in the presence or absence of precision biotic (PB). The concentrations of all measured SCFAs were increased by treatment with PB (*P* < 0.05), regardless of whether the source microbiome was from low BW or high BW birds.

Lastly, Figure 7 shows the concentration of acetic, propionic, and butyric acids in the supernatant obtained post *ex vivo* incubation of the cecal microbiome in the presence of PB. It was observed a significant (*P* < 0.05) enrichment in the concentration of all acids in the presence of PB, regardless of the BW of the animals from which the samples were collected. Additionally, propionic and butyric acids were not detected in the supernatant of the control group, but only in the presence of PB.

## DISCUSSION

The influx of glycans into the intestine, be it from endogenous or exogenous sources, is one major process that shapes the intestinal microbiome (Koropatkin et al., 2012). The mechanism by which Firmicutes bacteria acquire and utilize glycans is not completely understood, but it has been suggested that the ABC transporters, transmembrane or membrane-associated proteins, work together with glycoside hydrolases to perform carbohydrate degradation and uptake by the intestinal microbiome (Koropatkin et al., 2012). On the other hand, in Bacteroidetes, it has been shown that glycans bind to surface proteins and are broken down into large fragments by surface enzymes, transported into the periplasmic space, and hydrolyzed into simple sugars by specific enzymes, that are transported to the cytosol and activate HTCS-like regulator to induce expression of polysaccharide utilization genes (Hao et al., 2021). Therefore, the understanding of the mechanisms by which different intestinal bacteria utilizes dietary glycans is of paramount importance to precisely formulate nutritional strategies to harness the complete potential of the microbiome.

In the present studies, we sought to develop an index that could measure microbial metabolism of protein by investigating whether the dietary supplementation of a PB could enrich desirable metabolic pathways in the cecal microbiome and improve the growth performance of broiler chickens. Furthermore, we aimed to assess the effects of PB on the cecal microbiome obtained from broiler chickens with different BW using an *ex vivo* incubation model, and a functional metagenomic assay. Overall, we observed that the microbiome of heavier birds demonstrates a higher MPMI and that the supplementation of PB in the diets of broiler chickens also led to an increase in MPMI by shifting the microbiome metabolic functions toward desirable pathways related to nitrogen utilization, resulting in improved growth performance.

Additionally, we showed in the *ex vivo* assay that the incubation of the cecal microbiome with PB enriches host-beneficial microbiome pathways, regardless of whether the underlying microbiome was obtained from chickens with low or high BW, as measured by the increased quantitative MPMI, and the concentration of SCFA in the supernatant extracted after the incubation. These results strongly suggest that it is possible to modulate core intestinal microbiome functions through tailored dietary components independently of the host underlying intestinal microbial profile.

Walsh et al. (2021) and Jacquier et al. (2022) have reported the effects of the supplementation of this PB in broiler chickens. Walsh et al. (2021) showed, through a meta-analysis of 19 floor pen studies carried out under different conditions, the effect of 2 PB, referred as microbiome metabolic modulators 1 and 2 (**MMM1** and **MMM2**). It was observed that the MMM2, corresponding to the PB used by Jacquier et al. (2022) and in the present study, improved the FCR of broiler chickens by at least 3 points in 75% of the trials, and by 4 points in at least 63% of trials. The consistency reported by Walsh et al. (2021) is explained, at least in part, by the action of the PB on the microbiome, as reported by an increase in the abundance of genes related to propionate production (acrylate pathway), and nitrogen metabolism. Jacquier et al. (2022), besides improvements in the growth performance, observed that the PB improved litter and welfare characteristics, such as gait score. Accordingly, with the precise modulation of the microbiome metabolic pathways by PB, there will be positive outcomes beyond growth performance. For example, Blokker et al. (2022) showed that the supplementation of PB reduced the negative effects caused by coccidiosis in chickens by modulating the inflammatory responses.

We have pioneered advanced methods in whole genome sequencing and functional metagenomic analysis to measure the abundance of beneficial genes linked to protein metabolism. As presented herein, we developed a functional assay and an index that reflects microbiome metabolism of protein that bypasses digestion and becomes available to the microbial community in the caeca of chickens. It is known that undigested protein may be used by functional groups of bacteria, such as putrefactive or protein-fermenting bacteria, and can produce harmful metabolites to the host (Apajalahti and Vienola, 2016). For example, indoles are the toxic end-products of tryptophan fermentation that inhibit oxidative phosphorylation; tyrosine is converted to *p*-cresol and phenol that possesses similar toxicity as indoles, and ammonia when produced in excess and cannot be assimilated by the intestinal bacteria, is detrimental to several aspects of intestinal health (Apajalahti and Vienola, 2016). We demonstrated in the present study that through the action of PB, the microbiome metabolism was redirected toward a beneficial protein metabolism, as demonstrated by the higher MPMI in both *in vivo* and *ex vivo* assessments. This shows that beneficial end products may be released by the microbiome such as BCFA, SFCA, polyamines, and other amino acids as, for example, asparagine, produced from aspartate and ammonia through the action of the enzyme asparagine synthase (Hakim et al., 2021).

Nevertheless, the concentration of SCFA was increased in the presence of PB as shown in the *ex vivo* assay. Butyric acid has a broad range of effect in the intestine and systemically (Bortoluzzi et al., 2022). Acetate and propionate produced by the intestinal bacteria are absorbed and, in the liver, used as substrate for peripheral adipogenesis and gluconeogenesis, respectively (Sun et al., 2021). These SCFA are primarily produced from carbohydrates, but fermentation of amino acids may also occur (Krishnan et al., 2015). Taken together, the microbiome metabolic shift observed in the *in vivo* study, plus the observations from the *ex vivo* assay, may explain the improvement in growth performance obtained with the supplementation of PB.

In conclusion, PB supplementation in the diets of broiler chickens shifts the cecal microbiome pathways toward beneficial protein utilization, as measured by higher MPMI. Together these data support that 1) the microbiome of heavier birds possess a more efficient microbiome protein metabolism, and 2) the mode of action whereby PB enriches microbial metabolic functions responsible for protein metabolism and improves growth performance of broiler chickens. The *ex vivo* assessment demonstrated that the PB can modulate microbiome pathways regardless of the BW of the animals, as measured by higher abundance of genes related to positive outcomes, higher quantitative MPMI, and higher production of SCFA. We, therefore, anticipate that these methods could be used as markers of microbiome modulation in response to different dietary interventions. The functional metagenomic assay presented herein to assess targeted microbiome beneficial metabolic pathways also offers improved quantification with lower burden and cost than whole genome sequencing.
